# pH-Dependent Chiral Recognition of *D*- and *L*-Arginine Derived Polyamidoamino Acids by Self-Assembled Sodium Deoxycholate

**DOI:** 10.3390/polym12040900

**Published:** 2020-04-13

**Authors:** Federica Lazzari, Bruce D. Alexander, Robert M. Dalgliesh, Jenny Alongi, Elisabetta Ranucci, Paolo Ferruti, Peter C. Griffiths

**Affiliations:** 1 Dipartimento di Chimica, Università degli Studi di Milano, via C. Golgi 19, 20133 Milano, Italy; jenny.alongi@unimi.it (J.A.); elisabetta.ranucci@unimi.it (E.R.); 2 Faculty of Engineering and Science, University of Greenwich, Medway Campus, Chatham Maritime, Kent ME4 4TB, UK; b.alexander@gre.ac.uk; 3 ISIS Neutron and Muon Source, Science and Technology Facilities Council, Rutherford Appleton Laboratory, Rutherford Appleton Laboratory, Didcot, Oxfordshire OX11 0QX, UK; robert.dalgliesh@stfc.ac.uk

**Keywords:** polyamidoamino acid, chiral interactions, chiral polymers, sodium deoxycholate, enantiodiscrimination.

## Abstract

*D*- and *L*-arginine-based polyamidoamino acids, called *D*- and *L*-ARGO7, retain the chirality and acid/base properties of the parent α-amino acids and show pH-dependent self-structuring in water. The ability of the ARGO7 chiral isomers to selectively interact with chiral biomolecules and/or surfaces was studied by choosing sodium deoxycholate (NaDC) as a model chiral biomolecule for its ability to self-assembly into globular micelles, showing enantio-selectivity. To this purpose, mixtures of NaDC with *D*-, *L*- or *D,L*-ARGO7, respectively, in water were analysed by circular dichroism (CD) spectroscopy and small-angle neutron scattering (SANS) at different levels of acidity expressed in terms of pD and concentrations. Differences in the CD spectra indicated chiral discrimination for NaDC/ARGO7 mixtures in the gel phase (pD 7.30) but not in the solution phase (pD 9.06). SANS measurements confirmed large scale structural perturbation induced by this chiral discrimination in the gel phase yet no modulation of the structure in the solution phase. Together, these techniques shed light on the mechanism by which ARGO7 stereoisomers modify the morphology of NaDC micelles as a function of pH. This work demonstrates chirality-dependent interactions that drive structural evolution and phase behaviour of NaDC, opening the way for designing novel smart drug delivery systems.

## 1. Introduction

Chiral synthetic polymers may spontaneously self-organize into ordered structures, a property that has been exploited for applications in different fields, such as catalysis [1,2,3,4], drug-delivery [5,6], chiral recognition [7,8], chiral resolution [9,10,11], biosensing [12,13,14], and bioimaging [15].

An attractive design approach to provide synthetic polymers with unique optical properties is to introduce natural α-amino acids into either their main- or side-chain. Several examples of α-amino acid derived chiral polymers have been reported, many of which obtained by radical polymerization of *N*-acryloyl- or *N*-methacryloyl-amino acids [16,17,18]. In other cases, the α-amino acid residues are present as lateral substituents of a variety of polymers, such as for example polyacetylenes [19,20], polyolefins [21], poly(vinyl ethers) [22] and polyphosphazenes [23].

A different class of stimuli responsive polymers based on α-amino acids, named polyamidoamino acids (PAACs), has been obtained by the Michael-type polyaddition of α-amino acids with bisacrylamides, as for instance *N,N’*-methylenebisacrylamide [24,25,26,27,28,29,30], 2,2’-bis(acrylamidoacetic) acid [29,30,31], and 1,4-bis(acryloyl)piperazine [32]. In particular, the PAAC named *L*-ARGO7 was obtained from *N,N’*-methylenebisacrylamide and *L*-arginine [24]. *L*-ARGO7 proved highly cytobiocompatible (IC_50_ ≥ 8 mg mL^−1^) and was easily internalized in Balb/3T3 cells. *L*- and *D*-ARGO7, the latter prepared from *D*-arginine, exhibited pH-dependent circular dichroism (CD) spectra suggesting the existence of pH-sensitive conformations [25]. Molecular dynamic studies on *L*-ARGO7 shed light on these conformations, whose relevant characteristic was the similarity with the “hairpin” motif of proteins [25]. This characteristic was shared by other PAACs prepared from *L*-alanine, *L*-valine, *L*-leucine [26]. Subsequently the PAAC derived from *L*-tryptophan [27] was also prepared. As a further development, the controlled synthesis of *L*-ARGO7 and its copolymers with *L*-alanine was performed [28]. 

Sodium deoxycholate (NaDC) is a component of bile salts, whose molecular structure is characterized by a rigid, slightly bent steroid structure and a short aliphatic side chain. In physiological environments, bile salts form micelles with a chiral surface (chiral micelles) due to the spatial concentration of chiral headgroups at the micelle surface [33,34,35,36] able to solubilize lipids and fat-based compounds [37]. As regards morphology, cholate-derived micelles are polydisperse [38]. Small-angle neutron scattering (SANS) of NaDC has revealed globular and rod-like structures with varying aggregation numbers, highly affected by concentration [39,40], ionic strength and pH [41]. Upon decreasing pH, these rods become more elongated until a thixotropic gel is formed [42]. Cholic acids and cholate-derived compounds are exploited in the formulation of poorly soluble drugs [43,44], and facilitating the cross-membrane transport of bioactive molecules [45]. In addition, NaDC chiral micelles proved able to separate chiral drugs or organic compounds in micellar electrokinetic capillary chromatography [46,47,48,49].

In the present study, NaDC has been chosen as a model chiral surface for assessing the ability of *D*- and *L*-ARGO7 of establishing chirality-dependent interactions. 

## 2. Materials and Methods 

### 2.1. Materials 

Solvents and reagents, unless otherwise indicated, were analytical-grade commercial products and used as received. *L*-arginine (≥98.5%), *D*-arginine (≥99%) and *D,L*-arginine (≥98.5%), sodium deoxycholate (≥97%), deuterium oxide (D_2_O, 99.9%), deuterium chloride (DCl, 99%) and sodium deuteroxide (NaOD, 40 wt % in D_2_O) were purchased from Sigma-Aldrich (Milano, Italy), while *N,N’*-methylenebisacrylamide (MBA, 96%) from Acros Organics (Milano, Italy). 0.3 M HCl and 0.3 M NaOH volumetric standard solutions were purchased from Fluka Analytics (Milano, Italy). Ultrapure water (18 MΩ cm^−1^), produced with a Millipore Milli-Q^®^ apparatus (Darmstadt, Hesse, Germany), was used to prepare all solutions, except where stated otherwise (D_2_O).

### 2.2. Synthesis of ARGO7 Isomers

The synthesis of ARGO7 isomers was carried out as previously described for *L*-ARGO7 [24]. Briefly, *L*-, *D*- or *D,L*-arginine (2.191 g, 12.45 mmol) was added to a dispersion of *N,N’*-methylenebisacrylamide (2.000 g, 12.45 mmol) in water (4.1 mL) under stirring. The resultant slurry was warmed under stirring to 50 °C, gradually becoming a homogeneous solution. The reaction mixture was kept in the dark at the same temperature with occasional shaking for 6 days. After this time, the mixture was acidified to pH 3.5 with 5% hydrochloric acid and ultrafiltered through a membrane with 100 kDa nominal cut-off. The passed-through portion was then further ultrafiltered through a membrane with 5 kDa nominal cut-off. Finally, the retained solution was freeze-dried to retrieve the sample as an off-white powder. The yields were in the range 88 →- 90%. The molecular weights (by Size Exclusion Chromatography, SEC) of *D*-, *L*- and *D,L*-ARGO7 were 7700 (PD 1.54), 6500 (PD 1.43) and 6800 (PD 1.48), respectively. ^1^H NMR and FTIR-ATR spectra and assignments are reported in Supplementary Materials (Figures S1 and S2 in Supplementary Materials).

### 2.3. Characterizations

Size exclusion chromatography (SEC) traces were obtained for all polymers with Toso-Haas TSK-gel G4000 PW and TSK-gel G3000 PW columns connected in series, using a Waters model 515 HPLC pump (Waters, Milano, Italy) equipped with a Knauer autosampler 3800 (Knauer, Bologna, Italy), a light scattering (670 nm), a viscometer Viscotek 270 dual detector (Malvern, Roma, Italy) and a refractive index detector (Waters, Model 2410, Milano, Italy). The mobile phase was a 0.1 M Tris buffer (pH 8.00 ± 0.05) solution with 0.2 M sodium chloride. Sample concentration: 20 mg mL^−1^; flow rate: 1 mL min^−1^; injection volume: 20 µL; loop size: 20 µL; column dimensions: 300 × 7.5 mm^2^. The instrument constants were determined using PEO 19 kDa as a narrow polymer standard. Before analysis, each sample was filtered through a 0.2 µm Whatman^TM^ syringe filter (Whatman^TM^, Kent, Maidstone, UK).

Circular dichroism (CD) spectra were recorded using a Chirascan spectrophotometer (Applied Photophysics Ltd., Surrey, UK) equipped with a Peltier temperature control system. Typically, CD signals were acquired at 25 °C by scanning in the 190–280 nm range, in rectangular quartz cells with 1 mm path-length. Each spectrum was the average of four measurements at 2.5 s time-per-point. Samples of NaDC (5 mg mL^−1^), *D*- and *L*-ARGO7 (0.5 mg mL^−1^) and NaDC/ARGO7 mixtures were prepared in 0.1 M NaCl solution using D_2_O. The pD was adjusted to 7.30 and 9.06 with 0.1 M DCl or 0.1 M NaOD aqueous solutions and measured by a combined Metrohm microelectrode (Metrohm, Runcorn, Cheshire, UK).

Small-angle neutron scattering (SANS) experiments were performed on the Larmor diffractometer at the ISIS Spallation Neutron Source, Rutherford Appleton Laboratory, Didcot, UK. A momentum transfer range defined by Q = (4π/λ) sin(θ/2) between 0.0035 and 0.7 Å^−1^ was obtained by using neutron wavelengths (λ) spanning from 0.9 to 13.5 Å. The samples were contained in either 1- or 2-mm path length, UV-spectrophotometer grade, quartz cuvettes (Hellma, GmBh, Jena, Germany) and mounted in aluminium holders on top of an enclosed, computer-controlled, sample chamber. All experiments were conducted at 25 °C. To maximise the scattering intensities - the so-called “contrast” - solutions of protiated polymer and/or NaDC in D_2_O were utilised. Experimental measuring times were approximately 50–60 min. All scattering data were (a) normalized for the sample transmission, (b) background corrected using a quartz cell filled with solvent (D_2_O), and (c) corrected for the linearity and efficiency of the detector response using the instrument specific software distributed as part of the Mantid framework [50].

## 3. Results and Discussion

### 3.1. Synthesis of D-, L-, and D,L-ARGO7

**Scheme 1:**
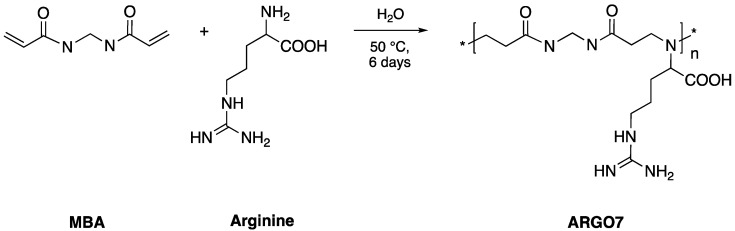
Synthesis of ARGO7.

*D*-, *L*, and *D,L*-ARGO7 were prepared in water by the Michael-type polyaddition of *D*-, *L*- or *D,L*-arginine with equimolar quantities of *N,N’*-methylenebisacrylamide (MBA) (Scheme 1), following a previously described procedure [25] here recalled in Materials and Methods. The weight-average molecular weights (*M*_w_) of all three stereoisomers were comparable. All samples were structurally characterized by ^1^H NMR (Figure S1). The spectra of all stereoisomers were superimposable and in agreement with the proposed structures.

### 3.2. Physico-Chemical Characterizations

To explore how the respective chirality of the polymer and NaDC affect the interaction between the two species, a number of experiments were performed varying the respective concentrations and charge on the polymer. The charge is controlled via the acidity of the solvent, which is expressed in terms of the pH when considering aqueous solutions. In the key experiments to be presented here, e.g., NMR, experimental protocols require the use of deuterated solvents, and therefore the acidity is expressed as pD. To minimise differences in pH and pD, where ever possible, D_2_O has been used as the solvent, even when it was not necessary, e.g., CD. 

#### 3.2.1. Phase Behaviour of NaDC/Water Mixtures

It is known that NaDC/water mixtures show a pH- [51] and concentration-dependent phase behaviour [52]. In order to identify the phase-stability boundaries of the chosen model system, 0.5–50 mg mL^−1^ NaDC/water mixtures in 0.1 M NaCl and at different pHs were studied in “normal” aqueous solutions.

These characterizations are presented in the Supplementary Materials. Briefly, when the NaDC concentration was higher than 2.5 mg mL^−1^, NaDC/water showed one of three phases: transparent homogenous liquids (pH ≥ 7.50), transparent homogenous gels (pH 7.0–7.5), homogenous but opalescent/”cloudy” gels (pH 6.5–7.0) (Figure S3 in supplementary materials), the latter associated with large structures commensurate with an approach to a phase boundary. Further decreases in pH lead to heterogenous systems, with insoluble material (“precipitate” in Figure S3). When the NaDC concentration was lower than 2.5 mg mL^−1^, only two phases were seen: transparent homogenous liquids (pH ≥ 6.50) and a heterogenous material with visible solid, termed “precipitate” (pH 6.00) (Figure S3). These heterogenous phases were not studied further. 

#### 3.2.2. Phase Behaviour of NaDC/Water/ARGO7 Mixtures

The isoelectric point of the ARGO7 stereoisomers is around 9.7 [24] and therefore, they are positively charged at pH < 9.0. Given that NaDC is negatively charged until –COOH neutralization, one would expect some level of pH-dependent electrostatic interactions in NaDC/water/ARGO7 mixtures and that such interactions may alter the phase transitions observed for the NaDC/water system. In addition, since *D*- and *L*-ARGO7, but not *D,L*-ARGO7, and NaDC are chiral, modification of the phase transitions may also occur due to any chirality-dependent interactions. Therefore, the stability and appearance of mixtures containing 0.5 wt % of either *D*-, *L*- or *D,L*-ARGO7 and 5 mg mL^−1^ NaDC in 0.1 M NaCl were also examined.

Differences were indeed observed for the gel phase and the onset of aggregation. In particular, for NaDC/water/*L*-ARGO7 mixtures, all samples at pH 7.60 formed a soft opaque gel compared with that obtained in the absence of the polymer. NaDC/water/*D*-ARGO7 mixtures gave a transparent gel at pH 7.50, whereas *D,L*-ARGO7 formed a transparent and harder gel at pH 7.25. These visible changes are clearly a reflection of some form of chiral interaction. Additionally, when the polymer was present in the mixtures, precipitation occurred at higher pHs than in the binary NaDC/water system, possibly associated to electrostatic interactions occurring between ARGO7 polymers and NaDC. As an example, in presence of *L*-ARGO7, precipitation occurred at pH 6.90 ± 0.10 instead than pH 6.70 ± 0.10.

#### 3.2.3. Additional Physico-Chemical Characterizations

Additional physico-chemical characterizations, such as surface tension (Figure S4 in supplementary materials), ζ-potential (Figures S5 and S6 in supplementary materials), dynamic light scattering (Table S1 in supplementary materials), and self-diffusion coefficients, *D_s_*, (Figures S7 and S8, Tables S2–S5 in supplementary materials) of NaDC/water/ARGO7 mixtures were also performed. The results obtained failed to reveal chiral interactions between *L*-ARGO7 and NaDC in these conditions.

#### 3.2.4. Circular Dichroism (CD) of NaDC/D_2_O/ARGO7 Stereoisomer Mixtures

The occurrence of NaDC conformational changes induced by pD-dependent chiral interactions with the ARGO7 stereoisomers was investigated by means of CD spectroscopy in 0.1 M NaCl/D_2_O solutions and two pD values, that is, pD 9.06, at which NaDC/D_2_O and NaDC/D_2_O/ARGO7 mixtures were homogeneous liquids, and pD 7.30, at which these systems were transparent gels. 

At pD 7.30, measurements were performed after 2 h, allowing the gel to reach full equilibrium. In fact, it was observed that gels obtained for high concentration samples (30 mg mL^−1^ of NaDC) gave reproducible and stable CD spectra after 2 h (Figure S9 in supplementary materials). 

Optimum conditions were informed by the underlying characterization presented in the Supplementary Materials—pD 9.06 and NaDC concentrations between 2.5–5.0 mg mL^−1^—these being characterized by a maximum transmittance value around 0.7 in the UV range 200–270 nm (Figure S10a in supplementary materials). This CD spectra showed a low intensity minimum at 211 nm (Figure S10b in supplementary materials). The major absorption, probably due to C=O groups, fell below 200 nm. As for ARGO7 isomers, the selected concentration was 0.5 mg mL^−1^, corresponding to a 10:1 NaDC/ARGO7 weight ratio, to minimize the electrostatic interactions (Figure S6).

**Figure 1:**
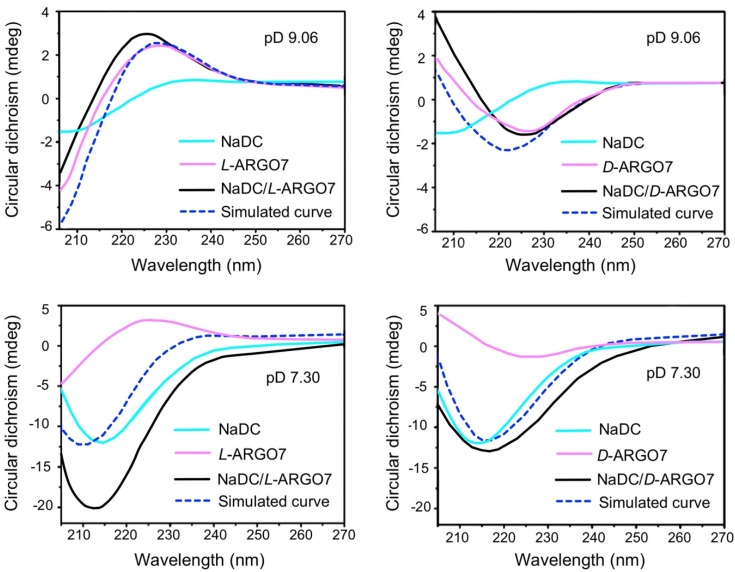
Comparison between the simulated and experimental circular dichroism (CD) spectra of NaDC/D_2_O/ARGO7 isomer mixtures at pD 7.30 and 9.06. Experimental conditions: NaDC concentration 5 mg mL^–1^; ARGO7 concentration 0.5 mg mL^–1^; temperature 25 °C; optical path 1 mm.

The CD spectra of the NaDC/D_2_O/*D*-ARGO7 and NaDC/D_2_O/*L*-ARGO7 mixtures at pD 9.06 and 7.30 are reported in Figure 1, together with the simulated traces (dotted blue line) calculated as the sum of the individual 5 mg mL^−1^ NaDC and 0.5 mg mL^−1^ ARGO7 CD traces.

If there are no interactions between the two components, one would expect that the simulated curve would reasonably closely resemble the experimental one. At pD 9.06, that is, when the NaDC/water/ARGO7 mixtures are transparent liquids, the simulated and experimental traces for both NaDC/D_2_O/L-ARGO7 and NaDC/D_2_O/D-ARGO7 mixtures do indeed show a significant similarity with respect to the related experimental curves (Figure 1), in other words, no traces of chiral-dependent interactions were detected. At pD 7.30, when the mixtures present as transparent gels, a different pattern is exhibited—the experimental trace and simulated curve for D-ARGO7/NaDC are very similar, but they differ markedly for the L-ARGO7/NaDC pairing (Figure 1), illustrating that chiral-dependent interactions exist.

#### 3.2.5. Small-Angle Neutron Scattering (SANS) Measurements: NaDC/D_2_O Systems

##### (i) Simple NaDC Solutions and Gels

In order to understand how the presence of a chiral polymer might modify the morphology of the model chiral substrate necessitates an understanding of the model chiral substrate itself. Therefore, a detailed investigation of the structure of NaDC micelles was carried out by means of small-angle neutron scattering (SANS). The effects of NaDC concentration and pH were principally explored and reported as scattering intensity vs. scattering vector (Q) with their pD.

**Figure 2:**
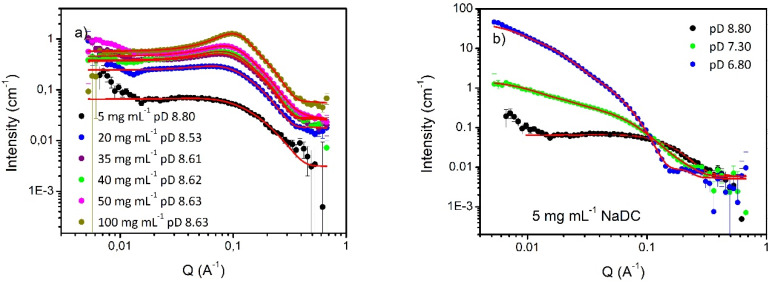
Small angle-neutron scattering data of NaDC as a function of: panel (**a**) concentration, at pD 8.5–8.8 and panel (**b**) pD, at 5 mg mL^−1^ concentration. Fittings are reported in the graph as red lines.

At pD 8.5–8.8, scattering data collected in the NaDC concentration range of 5–100 mg mL^−1^ showed curves consistent with the presence of micellar structures (Figure 2a), characterized by (largely) monotonic decays within this Q range at low concentrations. With increasing concentration, there is an increase in scattering intensity over the entire Q range, but the obvious emergence of a peak around Q = 0.15 Å^−1^ associated with the correlation in the micelle separation, i.e., interparticle interactions. In order to determine the micelle shape, all curves were fitted with the literature-informed [40] theoretical scattering function for prolate ellipsoids with polar radius of 22–28 Å and equatorial radius of 9–11 Å. To account for interparticle interactions between charged spheroidal objects, the Hayter–Penfold (HP) Rescaled Mean Spherical Approximation (RMSA) structure factor S(Q) was included in all model fitting. The HP S(Q) is an oscillatory function that reflects the relative position of the scattering bodies within the solution, and is parameterised through the volume fraction and size of the scatterers, their charge and the prevailing ionic strength of the medium [53,54]. Practically, for moderately concentrated surfactant systems, this function leads to peaks within the mid-Q range of a typical scattering curve, but for dilute cases, S(Q) = 1. As may be seen, the data are well-described by this model over the majority of the Q-range, except at very low Q, where the upturn in the intensity is consistent with a small population of larger structures. Note the emergence of the peak around Q ≈ 0.15 Å^−1^ associated with the HP S(Q). Fitted parameters are reported in Table S6 (supplementary materials) and were in all cases mutually comparable, indicating no significant morphological evolution over this range of sample parameter space. 

Upon decreasing pH, Figure 2b, there are significant changes in scattering pattern, reflective of the fact that the structure of NaDC micelles (at this concentration) changed from prolate ellipsoids (pD 8.80) to (much larger) elongated rods (pD 7.30), described by an elliptical cylinder model with length 490 ± 20 Å and axial ratio 2.9 ± 0.2 (Figure 2b and Table 1). This pH-dependent evolution of the rod-like micelle conformation is again, in good agreement with literature precedence [41]. Therefore, either the prolate ellipsoid or the elliptical cylinder model will be adopted in the ensuing data analysis. 

By further decreasing pH, on approaching the NaDC solubility phase boundary (pD 6.80), the scattering intensity again increased commensurate with longer rod dimensions, typically >650 ± 25 Å and axial ratio of 5.3 ± 0.3 (Figure 2b and Table 1). No other changes in the SANS pattern, i.e., NaDC conformation, were seen.

SANS collected in the 5*–*100 mg mL^−1^ range on NaDC/water mixtures in the transparent gel phase at pD 7.3*–*7.5 showed concentration-dependent structuring of NaDC. Results are reported in Supplementary Materials (Figure S11 in supplementary materials). Briefly, when NaDC concentration was lower than 35 mg mL^−1^ SANS pattern were always described by the elliptical cylinder model (Figure S11a). Interestingly, when the concentration was 40 and 50 mg mL^−1^, NaDC showed a layered structure with a peak centred at 0.07 Å^−1^ (Figure S11b). These SANS patterns suggested a layered structure composed of stacked rigid rods. The d-spacing describing the distance between scattering centres was calculated as Q = 2π/d and gave d = 9 nm. The same layered structure can still be seen at 100 mg mL^−1^ (Figure S11c), albeit with a less pronounced peak suggesting a more amorphous material than at the lowest concentrations. 

Thus, having verified the behaviour of the model chiral substrate, the scattering from simple 0.5 mg mL^−1^ polymer solutions was also examined. No measurable scattering was observed due no doubt to both the low size and concentration of the polymer. Consequently, it is assumed that for all the polymer/NaDC mixtures, the observed scattering is dominated by any NaDC/polymer complex, and that any “free” polymer may be neglected. No attempt is made here to distinguish the two components within the complex, rather the focus is on the gross morphology of the complex.

##### (ii) NaDC/ARGO7 Solutions and Gels

The scattering study was therefore focused on matrix of samples comprising 5 mg mL^−1^ NaDC and 0.5 mg mL^−1^ of *D-*, or *L-*, or *D,L*-ARGO7, or an equimolar mixture obtained by mixing *L-* and *D-*isomer of the polymer (*D*-/*L*-ARGO7) over a wide range of pD to explore the full phase behaviour exhibited by this system.

**Figure 3:**
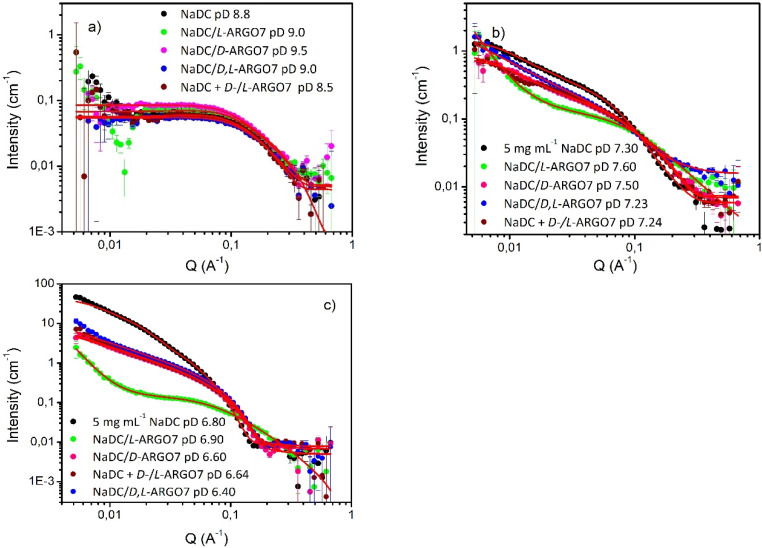
5 mg mL^−1^ NaDC scattering data in presence of 0.5 mg mL^−1^ *D*-, *L*-, *D,L*-ARGO7 and the equimolar mixture *D*-/*L*-ARGO7 at (**a**) pD 9.5*–*8.5; (**b**) pD 7.23*–*7.60; and (**c**) pD 6.40*–*6.90. Fittings are reported as red lines.

Consider first, mixtures prepared in the pD range of 8.50*–*9.50, conditions at which NaDC/water/ARGO7 systems resulted as transparent liquids (Figure S4), and where the circular dichroism data indicated an absence of any chiral interactions. Over this pD range, the SANS data were superimposable and were well-described by the same prolate ellipsoid model used for the NaDC/water system (Figure 3a) regardless of the stereoisomer, with only small changes in the fit parameters (Table 2). These results confirmed the conclusion observed from CD measurements, that is, the effect of any electrostatic interactions between ARGO7 stereoisomers and NaDC micelles, is modest and there is an absence of any chirality-dependent interactions.

However, and in good agreement with the CD characterization, significant changes in the scattering behaviour and therefore structures and inter alia the chiral interactions driving those changes, were observed at lower pDs, Figure 3b,c.

In the transparent gel phase with pD range of 7.30*–*7.50 NaDC/polymer mixtures (i.e., 5 mg mL^−1^ NaDC and 0.5 mg mL^−1^ of *D*-, *L*-, *D,L*-ARGO7, and equimolar blend of *D*-/*L*-ARGO7) showed not only an order of magnitude increase in scattering intensity, but quite marked changes in scattering pattern defined by the isomeric character of the polymer. Crucially, *D*-, *D,L*-ARGO7 and the equimolar mixture showed a mutually similar scattering pattern, and one that was highly reminiscent of the NaDC alone; i.e., in these mixtures, the rod-like structure of NaDC micelles is retained, albeit with a smaller radius and shorter length (Figure 3b and Table 2). The NaDC/*L*-ARGO7 dataset is strikingly different, and a two-correlation length model had to be deployed to describe this new morphology of the polymer/NaDC complex (Figure 3b and Table 3). Therefore, it is hypothesized that the presence of *L*-ARGO7 triggers the formation of clusters composed of NaDC micelles and polymer chains bound together by chiral interactions. These objects of higher dimensions are fitted by the first term of the model known as Porod scattering, whereas the second part, i.e., Lorentzian scattering, may be ascribed to the NaDC micellar component.

Chiral discrimination persists at even lower pD values, viz pD 6.40*–*6.90, when NaDC/water and NaDC/water/ARGO7 systems appeared as homogenous/opalescent gels. Similar to the previous case, there is a very pronounced increase (×10) in scattering intensity and again, *D*-, *D,L*-ARGO7 and the equimolar mixture gave rise to mutually similar patterns, but ones that were weaker in intensity to the simple NaDC case. All these particular datasets could be modelled by the elliptical cylinder model albeit with greater length and lower charge than NaDC/water over the same pD range (Figure 3c and Table 2). 

Once again, the *L*-ARGO7 case is quite different in shape and noticeably weaker in intensity (Figure 3c and Table 3). At pD 6.8, 30% of ARGO7 chains remain positively charged, while the remaining 70% were in their zwitterionic form, and able to induce H-bonding. It is assumed that electrostatic interactions between ARGO7 chains and NaDC neutralizes the carboxylate groups on the NaDC, causing precipitation at higher pH than NaDC/water alone and resulting in longer rods. Once the charge is neutralized, NaDC micelles pack together by hydrophobic forces, whose magnitude and orientation are shaped by the chirality of the polymer. 

##### (iii) The Effect of ARGO7 Chain Length on the NaDC/Water/ARGO7 Interaction

**Figure 4:**
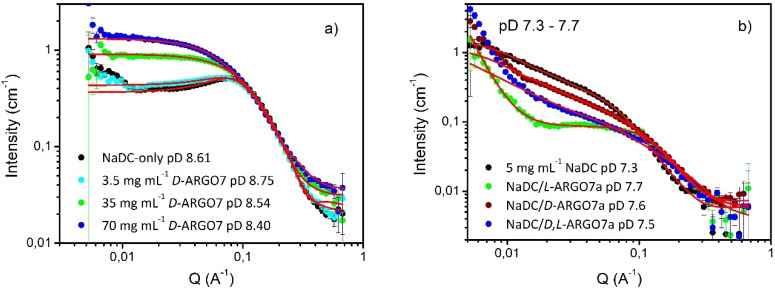
NaDC scattering data in presence of: panel (**a**) increasing concentration of *D*-ARGO7 and panel (**b**) *D*-, *L*-, *D,L*-ARGO7 with higher *M*_n_ (named *D*-, *L*- and *D,L*-ARGO7a). Fittings are reported as red lines.

In order to study if and how the length of the ARGO7 chains may affect the chiral-dependent NaDC self-structuring, chiral interactions were assessed in the NaDC/water/ARGO7 mixtures prepared with ARGO7a stereoisomers, named ARGO7, with higher number-average molecular weights, *M*_n_ *D*-ARGO7a = 8400, *M*_n_ *L-*ARGO7a = 10600 and *M*_n_ *D,L*-ARGO7a = 8800 (Figure 4b). Consistent with the insights on the previous polymers, the addition of *L*-ARGO7a triggered the same conformational change in NaDC micelle structure from ellipsoidal cylinders to clusters of complex morphology formed by NaDC micelles and polymer chains (Table 3). Instead, *D*- and *D,L*-ARGO7 retained the NaDC rod-like structures, albeit with higher lengths and smaller radius than NaDC/water (Table 2). In the ellipsoidal cylinder model used to fit NaDC mixtures with *D*- and *D,L*-ARGO7, high Q values associated with larger structures represented by the upturn in the graph, have been ignored.

These results suggest that an increase in the length (molecular weight) of the *L*-ARGO7 polymer chains has an effect on the morphology of the NaDC structures in aqueous solution, but the other isomers continue to have little impact.

##### (iv) The Effect of NaDC/ARGO7 Molar Ratio on the NaDC/Water/ARGO7 Interaction

In order to attempt to separate the effects of electrostatic interactions and chiral discrimination in the NaDC/water/ARGO7 liquid mixtures, an experiment focusing on the ratio of NaDC/*D*-polymer was conceived (Figure 4a and Table S7 in supplementary materials) as this would not be expected to show a chiral discrimination. There are polymer-concentration dependent changes in intensity, with the lowest concentration of polymer showing a negligible impact on the NaDC morphology, as one would expect given the forgoing conclusions. At higher polymer concentrations, scattering from the polymer will be become detectable, and the subtle change in form (the “disappearance” of the S(Q)) is merely reflective of its contribution, i.e., there appears to be no large-scale structural perturbation. Analysis of this dataset with the Gaussian coil model suggests *R*_G_ = 2.1 (± 0.2) nm, entirely consistent with the molecular weight of the polymer, and its self-diffusion coefficient (Tables S2–S3). 

At pD 8.40, the average ARGO7 net charge per repeating unit is slightly positive (+ 0.011): the *tert*-amine groups are almost completely deprotonated, the remaining internally neutralized by the carboxylate groups, whereas the guanidine pendant remains positively charged. Thus, with increasing ARGO7 concentration a decrease in the surface charge of NaDC is expected. When the NaDC/*D*-ARGO7 ratio ≤1, that is at 35 and 70 mg mL^−1^ of *D*-ARGO7 and 35 mg mL^−1^ NaDC, the addition of the polymer neutralized some of NaDC carboxylate groups and the object that is now causing scattering is composed of ellipsoidal NaDC and spherical ARGO7, in close proximity, due to the electrostatic interactions and the formation of hydrogen bonding (Figure 4a). The shape of the polymer and its surface charge are known from previous work [24,25]. In conclusion, when NaDC/*D*-ARGO7 ratio ≤ 1, SANS data confirmed the existence of electrostatic interactions between NaDC and *D*-ARGO7 in the liquid phase.

It has been shown that CD and SANS measurements are complementary tools to detect chiral recognition. Changes in CD spectra may now be associated with conformational modifications of both polymers and NaDC due to: (i) the concentration and pH-dependent self-assembly of both components into ordered chiral structures, whose morphology span from ellipsoidal to rod-like micelles in the case of NaDC; and ii) their chiral interaction. The latter caused the formation of a new object composed of NaDC and ARGO7 chains, of complex morphology. Hence, changes in the morphology of the scattering objects detected by SANS explain the modification seen in the CD pattern from the solution to the gel phase, in case of NaDC, and in presence of chiral interactions. 

## 4. Conclusions

Sodium deoxycholate (NaDC), one of the components of bile salts, was chosen as the chiral model surface to detect chiral interactions with ARGO7 stereoisomers. NaDC systems show three phases: homogenous solution (pH ≥ 7.50), homogeneous gel phase (pH 7.0–7.5), homogenous/opalescent gels leading to heterogenous systems (pH ≤ 7.00). The same phases were seen in the presence of 0.5 wt % ARGO7 stereoisomers, with some differences at higher NaDC concentrations being attributable to chiral interactions with the polymer. 

In the solution phase, all experimental methodologies *viz* surface tension, size, ζ-potential, PGSE-NMR measurements, CD and SANS all indicated that the presence of 0.5 wt % *L*-ARGO7 had no effect on the NaDC structure or dynamics, suggesting an absence of chiral interactions with NaDC in these conditions. In the gel phase, CD spectra of NaDC/polymer mixtures showed a divergence in comparator patterns—the presence of *L*-ARGO7 perturbed NaDC self-reorganization into an ordered gel, whereas *D*-ARGO7, *D,L*-ARGO7 did not i.e., *L*-ARGO7 triggered a morphological reorganization of NaDC. Again, in the solution phase, NaDC/water/ARGO7 stereoisomers mixtures showed superimposable scattering curves, indicating an absence of chiral discrimination, whereas in the gel phase, NaDC conformation was affected differentially by the chirality of the polymer. In particular, *D*- and *D,L*-ARGO7 changed the SANS pattern in the same way, retaining the rod-like structure of NaDC micelles, whereas *L*-ARGO7 induced the formation of NaDC ellipsoidal clusters. The same chiral-dependent interactions were observed at pD 6.40–6.90, close to the onset of insolubility, and at pD 7.50 for higher *M*_n_ ARGO7 polymers. Hence, chiral recognition seems to be a characteristic that is more pronounced within the gel phase, associated with the self-assembly of rod-like structures of NaDC.

From the CD and SANS results it can be concluded that *D*- and *L*-ARGO7 are able to discriminate, based on their chirality, a model chiral surface presented by NaDC micelles. This coupled with their already proved cytobiocompatibility and cell-internalization, may open the way for intracellular selective drug targeting or delivery of bioactive entities (proteins, liposomes and nucleotides to name a few).

## Figures and Tables

**Table 1:** Comparison between hydrated ellipsoid and the hydrated elliptical cylinder models for 5 mg mL^−1^ NaDC: pD-dependence of scattering length density (SLD), polar and equatorial radii and charge. All values have a standard deviation lower than 1%, except where indicated otherwise.

pD	Mathematical Model	SLD (10^−6^ Å^−2^)	*R*_minor_ (Å)	Axis Ratio	Length (Å)	*R*_pola*r*_ (Å)	*R*_equatorial_ (Å)	Charge (e)
8.80	Hydrated Ellipsoid	1.5 ± 0.1	-	-	-	23 ± 1	9	21 ± 3
7.30	Hydrated Elliptical Cylinder	1.3	11	3	490 ± 20	-	-	58 ± 2
6.80	Hydrated Elliptical Cylinder	1.4	23	5	630 ± 25	-	-	61 ± 1

**Table 2:** Parameters obtained for 5 mg mL^−1^ NaDC at various pD in presence of the polymers (*D*-, *L*-, *D,L*-ARGO7 and the equimolar mixture *D*-/*L*-ARGO7) using either the hydrated ellipsoid or the hydrated elliptical cylinder mathematical model. Standard deviation is not indicated when <1%. In all cases, scattering length density (SLD) values were between 2.1*–*2.3 10^-6^ Å^−2^.

pD	Polymer	*R*_minor_ (Å)	Axis Ratio	Length (Å)	*R*_polar_ (Å)	*R*_equatorial_ (Å)	Charge (e)
8.50*–*9.50	*L*-ARGO7	-	-	-	22 ± 1	8 ± 0.3	17 ± 3
	*D*-ARGO7	-	-	-	26 ± 1	9 ± 0.2	17 ± 2
	*D,L*-ARGO7	-	-	-	22 ± 1	9 ± 0.3	17 ± 3
	*D*-/*L*-ARGO7	-	-	-	22 ± 0.6	9 ± 0.3	25 ± 4
7.30*–*7.50	*D*-ARGO7	5 ± 0.6	2 ± 0.3	429 ± 24	-	-	44 ± 2
	*D,L*-ARGO7	5 ± 1	3 ± 1	505 ± 34	-	-	40 ± 3
	*D*-/*L*-ARGO7	9 ± 0.6	1 ± 0.2	540 ± 27	-	-	66 ± 2
7.30*–*7.60	*D*-ARGO7a^*^	7 ± 1	2	589 ± 27	-	-	35 ± 1
	*D,L*-ARGO7a^*^	5 ± 1	2	530 ± 30	-	-	50 ± 2
	*D*-/*L-*ARGO7a^*^	9 ± 1	3	550 ± 34	-	-	33 ± 1
6.60*–*6.90	*D*-ARGO7	14	2	> 650	-	-	10 ± 1
	*D,L*-ARGO7	16	2	> 650	-	-	4 ± 1
	*D*-/*L*-ARGO7	16	2	> 650	-	-	5 ± 1

* Parameters obtained in presence of *D*-, *D,L-, D-/L*-ARGO7a with higher *M*_n_.

**Table 3:** Parameters obtained for 5 mg mL^−1^ NaDC/water/*L*-ARGO7 system at various pD using the shape independent mathematical function: correlation length, Porod’s and Lorentz’s exponents. All values have a standard deviation lower than 1%.

pD	Polymer	Correlation Length (Å)	Porod’s Exponent	Lorentz’s Exponent (Å^−2^)
7.30*–*7.50	*L*-ARGO7	11	2.7	2.0
7.30*–*7.50	*L*-ARGO7a^*^	8	3.9 ± 0.1	3.6 ± 0.1
6.60*–*6.90	*L*-ARGO7	13	3.5	2.2

* Parameters obtained in presence of *L*-ARGO7a with higher *M*_n_.

## Supplementary Materials

The following are available online at https://www.mdpi.com/2073-4360/12/4/900/s1. Figure S1: ^1^H NMR spectrum of L-ARGO7 in D_2_O at pH 4.5 using a Brüker Avance III 400 MHz instrument. Asterisks represent the signals of methylene and double bond protons of the terminal acrylamide. Figure S2: FT-IR/ATR spectrum of *L*-ARGO7 recorded in the 4000 - 600 cm^−1^ range, 32 scans, 4 cm^−1^ resolution, at room temperature, by a PerkinElmer Frontier^®^ FT-IR/FIR spectrophotometer, equipped with a diamond crystal (penetration depth 1.66 mm). Figure S3: Phase behaviour of NaDC/water mixture as a function of pH and concentration. ★: transparent homogeneous liquid; ▲: transparent homogeneous gel; ◼: precipitate. Figure S4: Surface tension measurements of NaDC/water mixtures in 0.1 M NaCl, at pH 8.63 ± 0.47 and 24.7 ± 0.4 °C (black dots) before and after the addition of 0.5 wt % *L*-ARGO7 (grey dots). The CMC value is the cross-point of the two linear segments. Figure S5: NaDC/water mixtures trend of ζ-values with pH. Panel (a) NaDC concentration between 0.05–1.0 mg mL^−1^; panel (b) NaDC concentration between 2.5–5.0 mg mL^−1^. Data were collected in 0.1 M NaCl. Figure S6: pH-Dependence of the ζ-potential values of *L*-ARGO7 (black line), NaDC (grey line) and NaDC/water/*L*-ARGO7 mixtures (blue line) at NaDC concentrations higher than the CMC. *L*-ARGO7 concentration was 0.5 mg mL^−1^. Figure S7: Self-diffusion coefficients at pD 9 of: panel (a) NaDC in NaDC/water and NaDC in NaDC/water/*L*-ARGO7 systems; panel (b) *L*-ARGO7 in NaDC/water/*L*-ARGO7 systems. In both cases the *L*-ARGO7 concentration was 5 mg mL^−1^. For comparison purposes, in panel (b) the diffusion coefficient of plain *L*-ARGO7 in a 5 mg mL^−1^ aqueous solution is also reported. Figure S8: Self-diffusion coefficients (*D_s_*) at pD 7 - 8 of: panel (a) NaDC/water and NaDC/water/*L*-ARGO7 systems; panel (b) *L*-ARGO7 in the same mixtures. In this case, for comparison purposes, also 5 mg mL^−1^ *D_s_* of plain *L*-ARGO7 was reported (pale grey dotted line). Figure S9: CD spectra of 30 mg mL^−1^ NaDC/water systems in 0.1 M NaCl and pH 7.30 as a function of time. Figure S10: Concentration-dependence of: panel (a) UV-vis and panel (b) CD spectra of NaDC/water systems, recorded at pD 9.06 in quartz-cell of 1 mm path length. Figure S11: NaDC scattering data as a function of concentration, at pD 7.3 - 7.5. Mathematical fittings were reported as red lines. Table S1: Hydrodynamic radii of NaDC before and after adding *L*-ARGO7 from DLS volume size distribution. Table S2: Concentration dependence of *L*-ARGO7 self-diffusion coefficients and hydrodynamic radii in the pD range 4.5–5.0. Table S3: pD-Dependence of the *L*-ARGO7 self-diffusion coefficients and hydrodynamic radii at concentration 10 mg mL^−1^. Table S4: Hydrodynamic radii of NaDC alone and in the presence of *L*-ARGO7, at pD 9.0, obtained from PGSE-NMR applying Stokes-Einstein equation (Equation (2)). Table S5: Hydrodynamic radii of NaDC alone and in the presence of *L*-ARGO7, at pD 7–8, obtained from PGSE-NMR applying Stokes–Einstein equation (Equation (2)). Table S6: Parameters obtained for NaDC/water using the hydrated ellipsoid mathematical model: scattering length density (SLD), polar and equatorial radii and charge. Standard deviation is lower than 1%, except where indicated otherwise. Table S7: Parameters obtained for 35 mg mL^−1^ NaDC at pD 8.50–9.50 in the presence of 3.5, 35 or 70 mg mL^−1^ *D*-ARGO7: scattering length density (SLD), polar and equatorial radii and charge. All values have a standard deviation lower than 1%, except where stated otherwise.
