# Supplementary material for: pH-Dependent Chiral Recognition of D- and L-Arginine Derived Polyamidoamino Acids by Self-Assembled Sodium Deoxycholate
Source: Polymers (Basel). 2020 Apr 13;12(4):900. doi: 10.3390/polym12040900 (PMC7240376; doi:10.3390/polym12040900)
Supplement: Supplementary file 1 [file polymers-12-00900-s001.pdf]

SUPPLEMENTARY MATERIALS

pH-Dependent Chiral Recognition of *D*- and *L*-Arginine Derived Polyamidoamino acids by Self-assembled Sodium Deoxycholate

Federica Lazzari [1*], Bruce D. Alexander [2], Robert M. Dalgliesh [3], Jenny Alongi [1], Elisabetta Ranucci [1], Paolo Ferruti [1*] and Peter C. Griffiths [2*]

[1] Dipartimento di Chimica, Università degli Studi di Milano, via C. Golgi 19, 20133 Milano, Italy; jenny.alongi@unimi.it (J.A.); elisabetta.ranucci@unimi.it (E.R.);

[2] Faculty of Engineering and Science, University of Greenwich, Medway Campus, Chatham Maritime, Kent ME4 4TB, United Kingdom; Bruce D. Alexander b.alexander@gre.ac.uk (B.D.A.);

[3] ISIS Neutron and Muon Source, Science and Technology Facilities Council, Rutherford Appleton Laboratory, Rutherford Appleton Laboratory, Didcot, Oxfordshire OX11 0QX, UK; Robert M. Dalgliesh robert.dalgliesh@stfc.ac.uk (R.M.D.)

* Correspondence: federica.lazzari@unimi.it (F.L.); paolo.ferruti@unimi.it (P.F.); Tel.: +39–02-50314128 (P.F.); p.griffiths@greenwich.ac.uk (P.G.).

Pages: S1-S15

Figures S1-S11

Figure S1. ^1H NMR spectrum of *L*-ARGO7 in D_2O at pH 4.5 using a Brüker Avance III 400 MHz instrument. Asterisks represent the signals of methylene and double bond protons of the terminal acrylamide.

Figure S2. FT-IR/ATR spectrum of *L*-ARGO7 recorded in the 4000 - 600 cm^{-1} range, 32 scans, 4 cm^{-1} resolution, at room temperature, by a PerkinElmer Frontier® FT-IR/FIR spectrophotometer, equipped with a diamond crystal (penetration depth 1.66 μm).

Figure S3. Phase behaviour of NaDC/water mixture as a function of pH and concentration. ★: transparent homogeneous liquid; ▲: transparent homogeneous gel; ■: precipitate.

Figure S4. Surface tension measurements of NaDC/water mixtures in 0.1 M NaCl, at pH 8.63 ± 0.47 and 24.7 ± 0.4 °C (black dots) before and after the addition of 0.5 wt.-% *L*-ARGO7 (grey dots). The CMC value is the cross-point of the two linear segments.

Figure S5. NaDC/water mixtures trend of ζ-values with pH. Panel (a) NaDC concentration between 0.05 - 1.0 mg mL^{-1}; panel (b) NaDC concentration between 2.5 - 5.0 mg mL^{-1}. Data were collected in 0.1 M NaCl.

Figure S6. pH-Dependence of the ζ-potential values of *L*-ARGO7 (black line), NaDC (grey line) and NaDC/water/*L*-ARGO7 mixtures (blue line) at NaDC concentrations higher than the CMC. *L*-ARGO7 concentration was 0.5 mg mL^{-1}.

Figure S7. Self-diffusion coefficients at pD 9 of: panel (a) NaDC in NaDC/water and NaDC in NaDC/water/*L*-ARGO7 systems; panel (b) *L*-ARGO7 in NaDC/water/*L*-ARGO7 systems. In both cases the *L*-ARGO7 concentration was 5 mg mL^{-1}. For comparison purposes, in panel (b) the diffusion coefficient of plain *L*-ARGO7 in a 5 mg mL^{-1} aqueous solution is also reported.

Figure S8. Self-diffusion coefficients (D_s) at pD 7 - 8 of: panel (a) NaDC/water and NaDC/water/*L*-ARGO7 systems; panel (b) *L*-ARGO7 in the same mixtures. In this case, for comparison purposes, also 5 mg mL^{-1} D_s of plain *L*-ARGO7 was reported (pale grey dotted line).

Figure S9. CD spectra of 30 mg mL^{-1} NaDC/water systems in 0.1 M NaCl and pH 7.30 as a function

of time.

Figure S10. Concentration-dependence of: panel (a) UV-vis and panel (b) CD spectra of NaDC/water systems, recorded at pD 9.06 in quartz-cell of 1 mm path length.

Figure S11. NaDC scattering data as a function of concentration, at pD 7.3 - 7.5. Mathematical fittings were reported as red lines.

Tables S1-S7

Table S1. Hydrodynamic radii of NaDC before and after adding *L*-ARGO7 from DLS volume size distribution.

Table S2. Concentration dependence of *L*-ARGO7 self-diffusion coefficients and hydrodynamic radii in the pD range 4.5 - 5.0.

Table S3. pD-Dependence of the *L*-ARGO7 self-diffusion coefficients and hydrodynamic radii at concentration 10 mg mL^{-1}.

Table S4. Hydrodynamic radii of NaDC alone and in the presence of *L*-ARGO7, at pD 9.0, obtained from PGSE-NMR applying Stokes-Einstein equation (Equation (2)).

Table S5. Hydrodynamic radii of NaDC alone and in the presence of *L*-ARGO7, at pD 7 - 8, obtained from PGSE-NMR applying Stokes-Einstein equation (Equation (2)).

Table S6. Parameters obtained for NaDC/water using the hydrated ellipsoid mathematical model: scattering length density (SLD), polar and equatorial radii and charge. Standard deviation is lower than 1%, except where indicated otherwise.

Table S7. Parameters obtained for 35 mg mL^{-1} NaDC at pD 8.50 - 9.50 in the presence of 3.5, 35 or 70 mg mL^{-1} *D*-ARGO7: scattering length density (SLD), polar and equatorial radii and charge. All values have a standard deviation lower than 1 %, except where stated otherwise.

Reference

1. Stilbs P., Paulsen K., Griffiths P. C. Global least-squares analysis of large, correlated spectral data sets: application to component-resolved FT-PGSE NMR spectroscopy. *J. Phys. Chem.* **1996**, *100*, 8180-8189, doi:10.1021/jp9535607.

^{1}H NMR and FT-IR/ATR Measurements

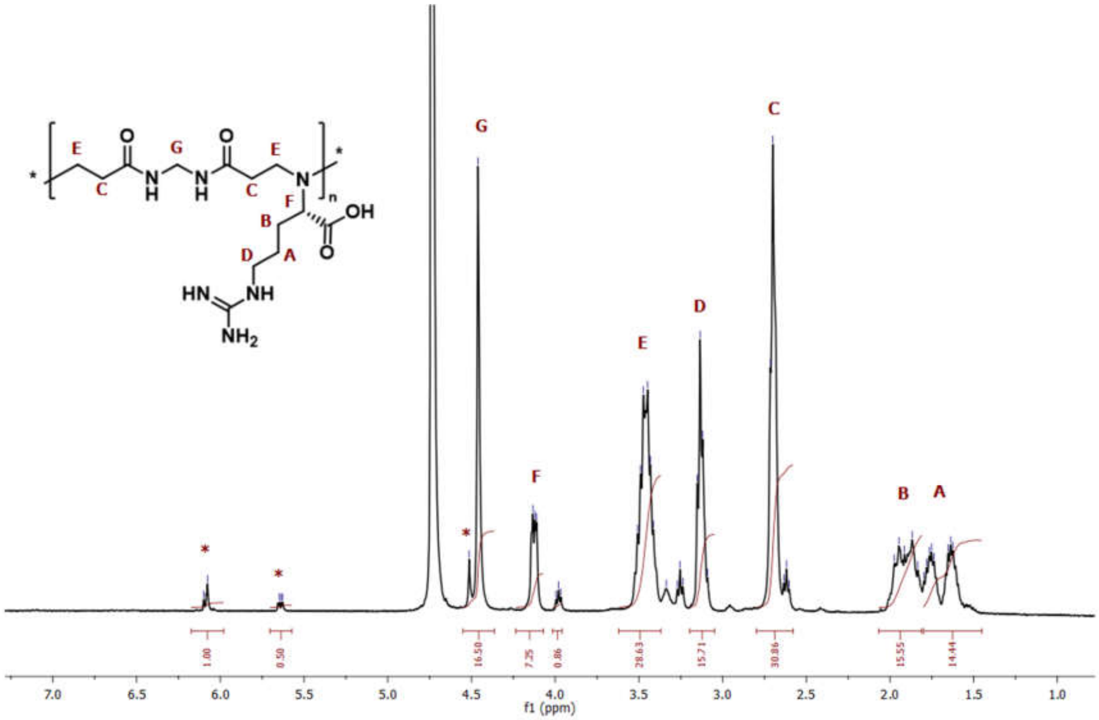

Figure S1. ^{1}H NMR spectrum of *L*-ARGO7 in D_2O at pH 4.5 using a Brüker Avance III 400 MHz instrument (Brüker, Coventry, West Midlands, UK). Asterisks represent the signals of methylene and double bond protons of the terminal acrylamide.

^{1}H NMR (D_2O, 400.132 MHz, ppm): 1.62-1.80 (m, 2H, C**H**$_2$CH$_2$CHCOO-), 1.83-1.97 (m, 2H, CH$_2$C**H**$_2$CHCOO-), 2.70-2.71 (m, 4H, COC**H**$_2$CH$_2$N), 3.09-3.15 (m, 2H, C**H**$_2$CH$_2$CH$_2$CHCOO-), 3.41-3.51 (m, 4H, COCH$_2$C**H**$_2$N), 4.11-4.13 (m, 1H, CH$_2$C**H**COO−), 4.46 (s, 2H, NHC**H**$_2$NH), 4.51 (s, 2H, NHC**H**$_2$NH of terminal acrylamide), 5.63-5.65 and 6.08-6.10 ppm (m, 3H, **H**$_2$C=C**H** of terminal acrylamide).

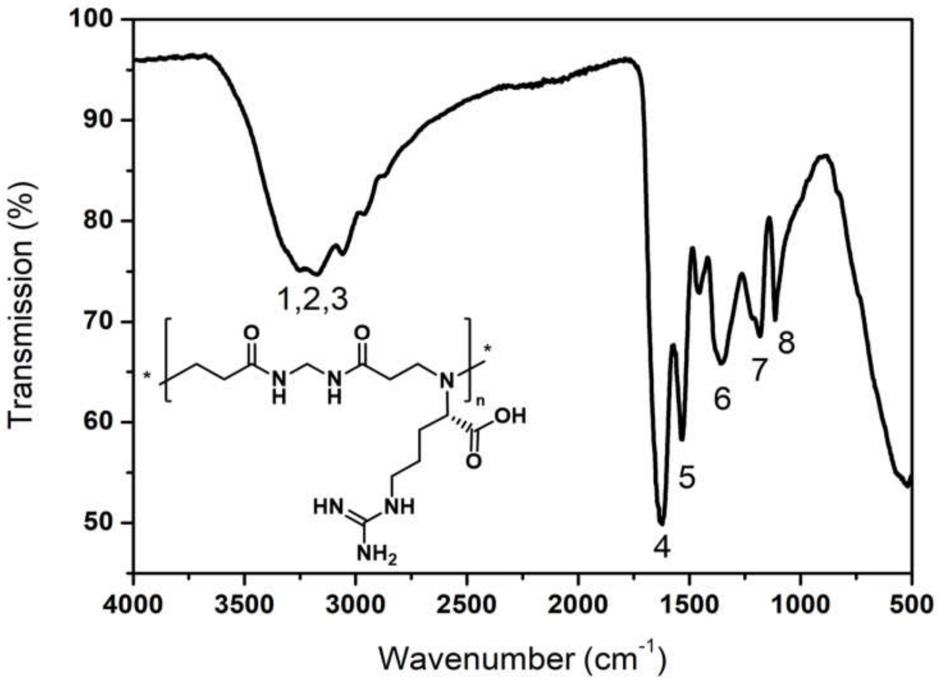

Figure S2. FT-IR/ATR spectrum of *L*-ARGO7 recorded in the 4000 - 600 cm^{-1} range, 32 scans, 4 cm^{-1} resolution, at room temperature, by a PerkinElmer Frontier® FT-IR/FIR spectrophotometer (Perkin Elmer, Milano, Italy), equipped with a diamond crystal (penetration depth 1.66 μm) .

Peak assignments: 3276 cm^{-1} (signal 1, O-H stretching); 3180 cm^{-1} (signal 2, N-H stretching); 2954 cm^{-1}, (signal 3, C-H stretching); 1641 cm^{-1} (signal 4, C=O stretching); 1536 cm^{-1} (signal 5, N-H bending); 1387 cm^{-1} (signal 6, O-H bending); 1187 cm^{-1} (signal 7, C-N stretching); 1115 cm^{-1} (signal 8, C-O stretching).

NaDC/water system phase behaviour

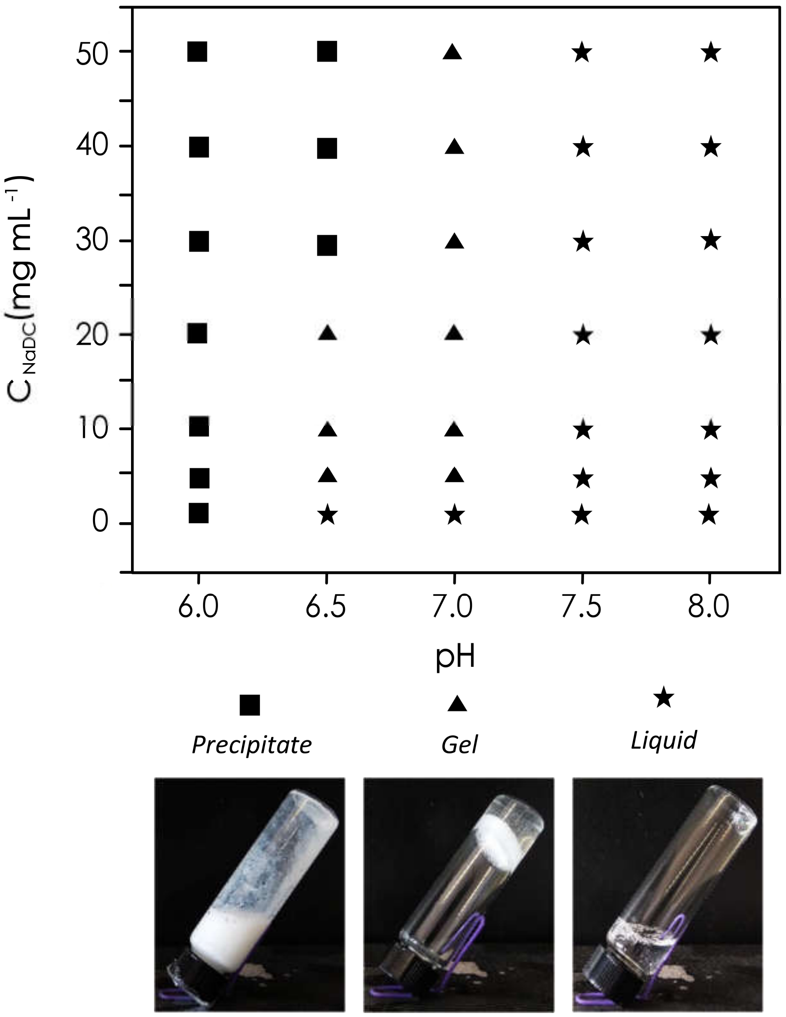

Figure S3. Phase behaviour of NaDC/water mixture as a function of pH and concentration. ★: transparent homogeneous liquid; ▲: transparent homogeneous gel; ■: precipitate.

Surface Tension Measurements

The surface tension of aqueous surfactant/polymer solutions was measured in triplicate at 25°C using a maximum bubble pressure tensiometer (SITA Science online t60 made by Sita Messtechnik GmbH, Dresden, Germany), calibrated with deionized water. A bubble lifetime of 15 s was used to ensure full equilibration. The surface tension measurements were carried out on NaDC/water and NaDC/water/*L*-ARGO7 mixtures in 0.1 M NaCl at pH > 8 (8.60 and 8.80, respectively) and 24.7 ± 0.4 °C. The critical micelle concentration (CMC) of NaDC in the absence and in the presence 0.5 wt.-% of *L*-ARGO7 was determined and showed that NaDC and *L*-ARGO7 did not interact.

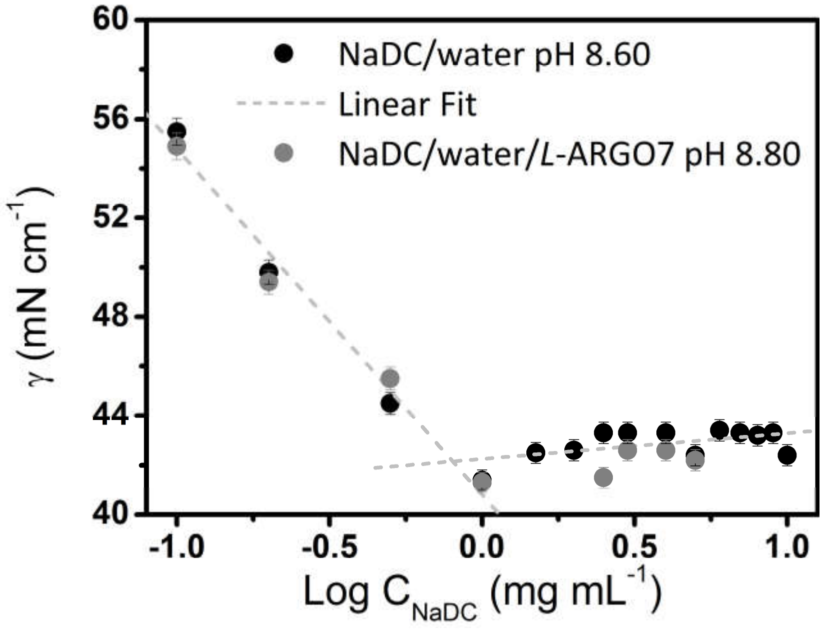

Figure S4. Surface tension measurements of NaDC/water mixtures in 0.1 M NaCl, at pH 8.63 ± 0.47 and 24.7 ± 0.4 °C (black dots) before and after the addition of 0.5 wt.-% *L*-ARGO7 (grey dots). The CMC value is the cross-point of the two linear segments.

ζ-Potential Measurements

ζ-potentials measurements were carried out in a folded capillary cells (0.5 mm) on NaDC/*L*-ARGO7 aqueous solutions by a Malvern Zetasizer NanoZS instrument (Malvern, Worcestershire, UK). This instrument was equipped with a 532 nm laser and fixed 173° scattering angle. The solution pH was adjusted to the selected value by the MPT-2 autotitrator (Malvern, Worcestershire, UK), using 0.3 M HCl or 0.3 M NaOH aqueous solutions. Before each measurement, samples were filtered through a 0.2 μm Whatman™ syringe filter (Maidstone, Kent, UK). Data were collected in Monomodal Mode, once the stability of the *ζ*-potential was reached. Values were reported as an average of 6 runs. *ζ*-potential measurements were carried out on both NaDC/water and NaDC/water/*L*-ARGO7 mixtures in 0.1 M NaCl and pH 6.0 - 9.5. NaDC concentration was 2.5, 3.5, 4.5 and 5.0 mg mL^{-1} and the concentration of *L*-ARGO7 was 0.5 mg mL^{-1}. The *ζ*-potential values suggested absence of interactions in these conditions, in agreement with surface tension measurements.

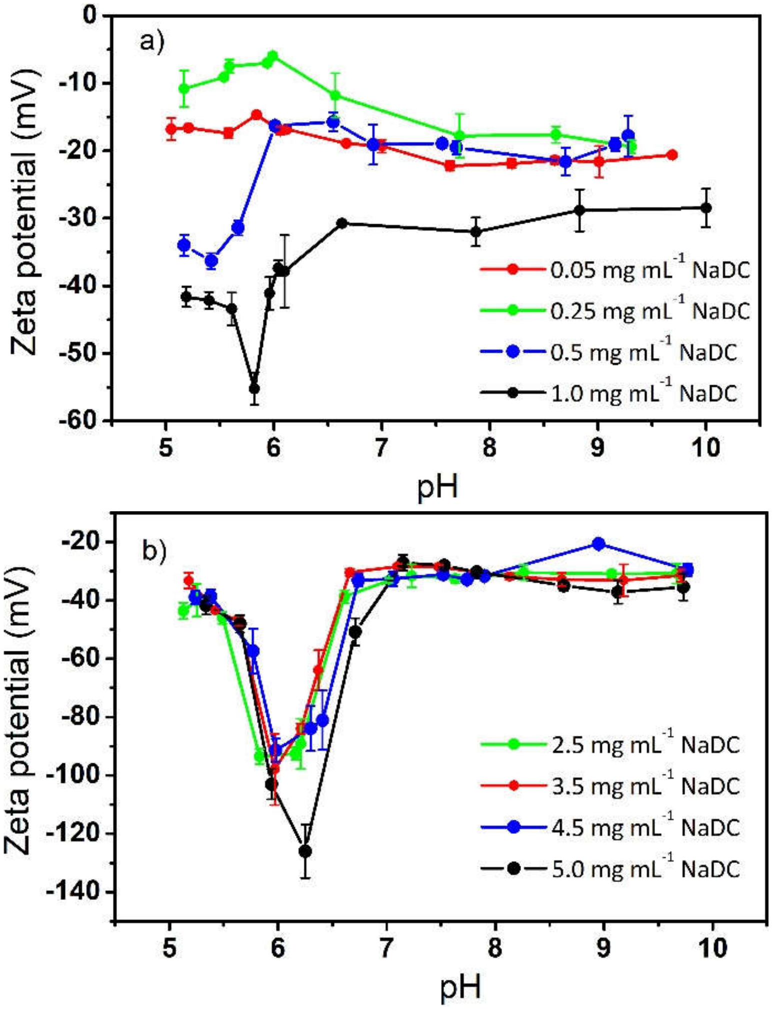

Figure S5. NaDC/water mixtures trend of ζ-values with pH. Panel (a) NaDC concentration between 0.05 - 1.0 mg mL^{-1}; panel (b) NaDC concentration between 2.5 - 5.0 mg mL^{-1}. Data were collected in 0.1 M NaCl.

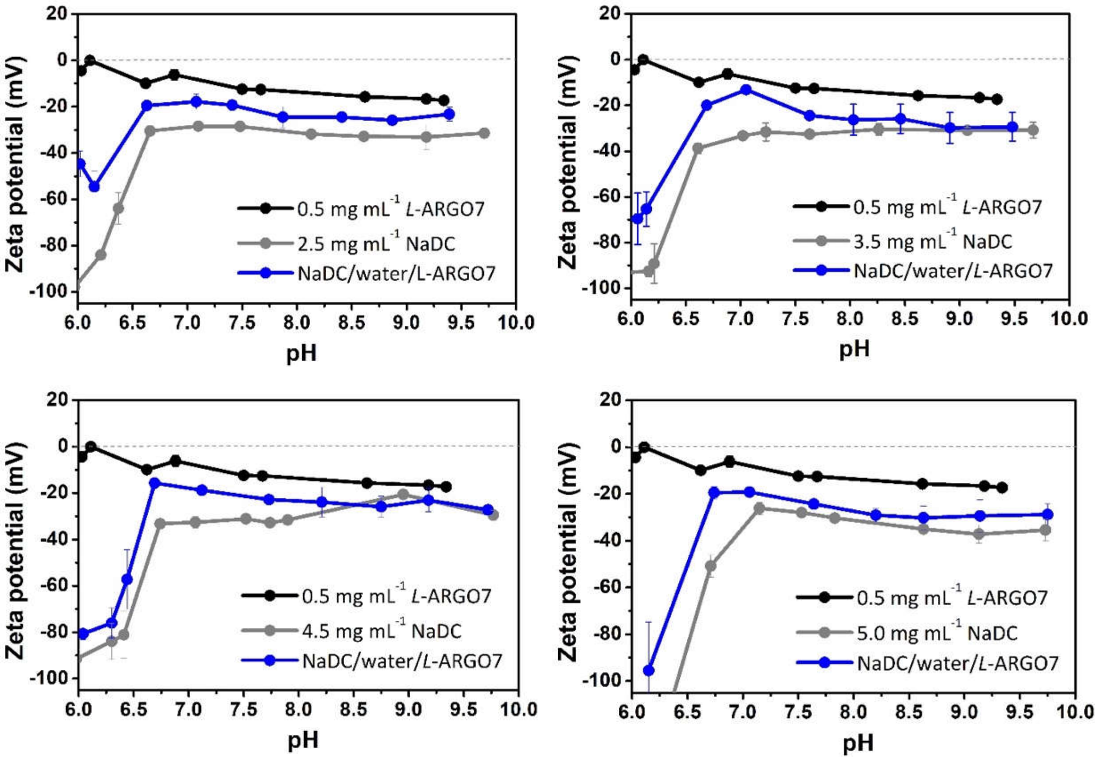

Figure S6. pH-Dependence of the ζ-potential values of *L*-ARGO7 (black line), NaDC (grey line) and NaDC/water/*L*-ARGO7 mixtures (blue line) at NaDC concentrations higher than the CMC. *L*-ARGO7 concentration was 0.5 mg mL^{-1}.

Dynamic Light Scattering Measurements

Dynamic Light Scattering (DLS) analyses were carried out on several aqueous solutions of NaDC and *L*-ARGO7 at different concentrations, using 0.1 M NaCl, by a Malvern Zetasizer NanoZS instrument (Malvern, Worcestershire, UK). This instrument was equipped with a 532 nm laser and fixed 173° scattering angle. Before each analysis, all samples were filtered through a 0.2 μm Whatman™ syringe filter (Maidstone, Kent, UK). The solution pH was adjusted to the selected value by the MPT-2 autotitrator (Malvern, Worcestershire, UK), using 0.3 M HCl or 0.3 M NaOH aqueous solutions. Typically, measurements were performed in triplicate and each value reported as the average of 10 runs. DLS measurements were carried out on both NaDC/water and NaDC/water/*L*-ARGO7 mixtures in 0.1 M NaCl and pH ≥ 7.50. NaDC concentration was 2.5, 3.5, 4.5 and 5.0 mg mL^{-1} and the concentration of *L*-ARGO7 was 0.5 mg mL^{-1}. The hydrodynamic radii (R_h) suggested absence of interactions in these conditions.

Table S1. Hydrodynamic radii of NaDC before and after adding *L*-ARGO7 from DLS volume size distribution.

	**NaDC/water**	**NaDC/water/*L*-ARGO7**[a]
C_{NaDC} **(mg mL^{-1})**	R_h **NaDC (nm) at pH 8.50**	R_h **NaDC (nm) at pH 8.50**
2.5	1.32 ± 0.29	1.83 ± 0.49
3.5	1.70 ± 0.45	1.63 ± 0.46
4.5	1.39 ± 0.41	1.50 ± 0.40
5.0	1.63 ± 0.37	1.57 ± 0.46

[a] In all cases, the concentration of *L*-ARGO7 was 0.5 mg mL^{-1} and its size 2.05 ± 0.53 nm, at pH 7.5.

Self-Diffusion coefficients by PGSE-NMR measurements [1]

Pulsed-gradient spin-echo nuclear magnetic resonance (PGSE-NMR) was recorded in D_2O at 25 °C using a 400 MHz Brüker FT NMR spectrometer (Coventry, West Midlands, UK) operating at 400.13 MHz, employing a 5 mm diffusion probe. Spectra were recorded at different pD, using diluted NaOD and DCl solutions.

PGSE-NMR experiment was carried out to determine the self-diffusion coefficients, D_s, of NaDC, *L*-ARGO7 and their mixtures. A stimulated echo sequence was used, in which the diffusion time (Δ) was set to 150 ms, the duration of the gradient pulses (δ) was held constant at 1 ms with intensities (G) varying from 5 to 300 G cm^{-1}. The number of scans was set to 16, accumulated over 32 gradient steps. Typically, self-diffusion coefficients (D_s) were calculated with CORE software, which fits peak intensities (I) to Equation 1 assuming a number of components present in the sample. For the majority of the experiments here, this was either 1 (polymer only) or 2 (polymer and NaDC).

$$I = I_0 e^{-D_s \gamma^2 G^2 \delta^2 (\Delta - \frac{\delta}{3})} \quad (1)$$

I_0 is the signal intensity in the absence of gradient pulses and γ the gyromagnetic ratio of protons [1]. The hydrodynamic radii, R_h, were obtained from the D_s values using the Stokes-Einstein equation, Equation (2)

$$D = k_B T / 6\pi\eta R_h \quad (2)$$

where k_B is the Boltzmann's constant, T the absolute temperature, η the solvent viscosity, and R_h the hydrodynamic radius of the particle. Measurements were carried out in D_2O containing 0.1 M NaCl solutions of NaDC, *L*-ARGO7 and their mixtures. A matrix of experiments was conducted to explore the effects of concentration, pH and ionic strength. In the case of *L*-ARGO7, concentrations in the 5 - 30 mg mL^{-1} range and pDs of 2.0, 5.0, 7.0, 9.0, and 11.0 were investigated. Whereas, in the case of NaDC concentrations were in the 0.5 - 50 mg mL^{-1} range and pDs of 7-8 and 9.0. NaDC/water/*L*-ARGO7 mixtures were tested at the following conditions: NaDC concentration in the 0.5 - 50 mg mL^{-1} range and *L*-ARGO7 concentration of 5 mg mL^{-1}, at pD of 7 - 8 and 9.0.

Results suggested that NaDC and *L*-ARGO7 either did not interact in these conditions or that an interaction occurs but does not lead to change in size. Indeed, in all cases, the D_s of NaDC was not significantly affected by the presence of *L*-ARGO7. The same holds true for *L*-ARGO7, whose size did not change with increasing NaDC concentration.

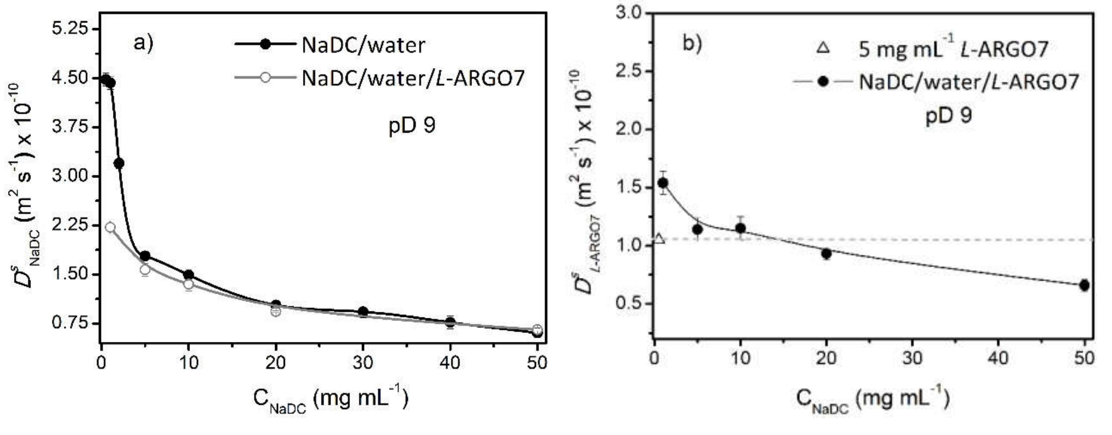

Figure S7. Self-diffusion coefficients at pD 9 of: panel (a) NaDC in NaDC/water and NaDC in NaDC/water/*L*-

ARGO7 systems; panel (b) *L*-ARGO7 in NaDC/water/*L*-ARGO7 systems. In both cases the *L*-ARGO7 concentration was 5 mg mL^{-1}. For comparison purposes, in panel (b) the diffusion coefficient of plain *L*-ARGO7 in a 5 mg mL^{-1} aqueous solution is also reported.

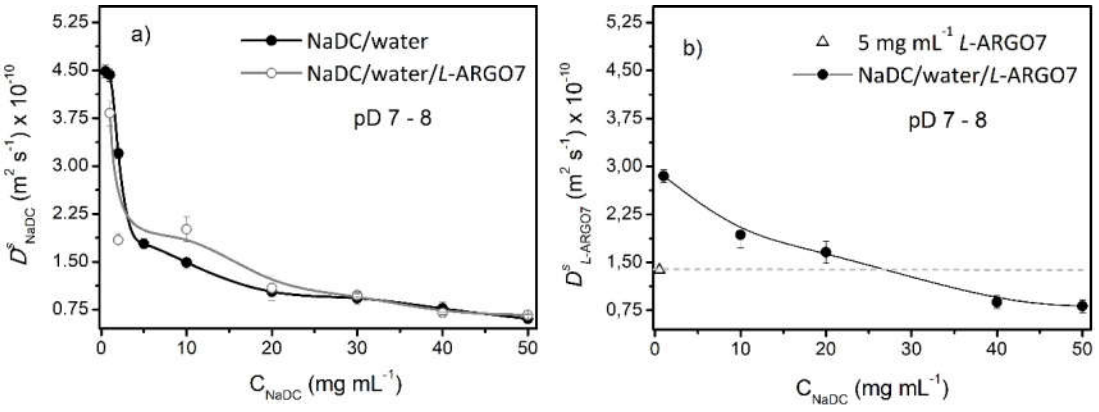

Figure S8. Self-diffusion coefficients (D_s) at pD 7 - 8 of: panel (a) NaDC/water and NaDC/water/*L*-ARGO7 systems; panel (b) *L*-ARGO7 in the same mixtures. In this case, for comparison purposes, also 5 mg mL^{-1} D_s of plain *L*-ARGO7 was reported (pale grey dotted line).

D_s values obtained for pure components, *L*-ARGO7 and NaDC (Tables S2-S5) were in line with those previously obtained by DLS. The same D_s results were obtained for *D*- and *D,L*-ARGO7 as a function of pD and polymer concentration.

Table S2. Concentration dependence of *L*-ARGO7 self-diffusion coefficients and hydrodynamic radii, in the pD range 4.5 - 5.0.

**C** (mg mL^{-1})	D_s ($m^2 s^{-1}$ x 10^{-10})	R_h (nm)
5	1.11 ± 0.02	1.93 ± 0.07
10	0.876 ± 0.04	2.45 ± 0.16
15	0.942 ± 0.02	2.28 ± 0.08
20	0.827 ± 0.04	2.60 ± 0.16
30	0.872 ± 0.03	2.46 ± 0.12

Table S3. pD-Dependence of the *L*-ARGO7 self-diffusion coefficients and hydrodynamic radii at concentration 10 mg mL^{-1}.

**pD**	D_s ($m^2 s^{-1}$ x 10^{-10})	R_h (nm)
2.0 ± 0.1[a]	0.757 ± 0.04	2.84 ± 0.03
5.0 ± 0.4	0.899 ± 0.10	2.39 ± 0.12
7.0 ± 0.2[a]	1.38 ± 0.09	1.56 ± 0.19
11.0 ± 0.8	1.18 ± 0.09	1.82 ± 0.18

[a] Self-diffusion coefficients were extracted from the Equation (1) due to the small intensity of each proton signal.

Table S4. Hydrodynamic radii of NaDC alone and in the presence of *L*-ARGO7, at pD 9.0, obtained from PGSE-NMR applying Stokes-Einstein equation (Equation (2)).

	**NaDC/water**	**NaDC/water/*L*-ARGO7**	**NaDC/water/*L*-ARGO7**
C_{NaDC} (mg mL^{-1})	R_h **NaDC (nm)**	R_h **NaDC (nm)**	R_h ***L*-ARGO7 (nm)**
0.5	0.480 ± 0.20	-	-
1	0.484 ± 0.09	0.970 ± 0.18	1.40 ± 0.20
2	0.671 ± 0.12	-	-
5	1.20 ± 0.35	1.37 ± 0.18	1.89 ± 0.33
10	1.44 ± 0.21	1.59 ± 0.15	1.87 ± 0.28
20	2.08 ± 0.32	2.31 ± 0.30	2.35 ± 0.36
30	2.31 ± 0.33	-	-
40	2.80 ± 0.39	-	-
50	3.53 ± 0.27	3.28 ± 0.44	3.26 ± 0.33

Table S5. Hydrodynamic radii of NaDC alone and in presence of *L*-ARGO7, at pD 7 - 8, obtained from PGSE-NMR applying Stokes-Einstein equation (Equation (2)).

	**NaDC/water**	**NaDC/water/*L*-ARGO7**	**NaDC/water/*L*-ARGO7**
C_{NaDC} (mg mL^{-1})	R_h **NaDC (nm)**	R_h **NaDC (nm)**	R_h ***L*-ARGO7 (nm)**
0.5	0.480 ± 0.20	-	-
1	0.484 ± 0.09	0.560 ± 0.12	0.75 ± 0.19
2	0.671 ± 0.12	-	-
5	1.20 ± 0.35	0.99 ± 0.21	-[a]
10	1.44 ± 0.21	1.07 ± 0.20	1.11 ± 0.17
20	2.08 ± 0.32	1.97 ± 0.33	1.29 ± 0.21
30	2.31 ± 0.33	2.20 ± 0.27	-[a]
40	2.80 ± 0.39	3.08 ± 0.36	2.44 ± 0.33
50	3.53 ± 0.27	3.22 ± 0.32	2.63 ± 0.29

[a] In this cases CORE software gave only one D_s value associated to either NaDC, *L*-ARGO7 or both. In no case two distinct values of D_s, for NaDC and *L*-ARGO7, were obtained.

Circular Dichroism (CD) Measurements

Circular dichroism (CD) spectra were recorded using a Chirascan spectrophotometer (Applied Photophysics Ltd., Surrey, UK) equipped with a Peltier temperature control system.

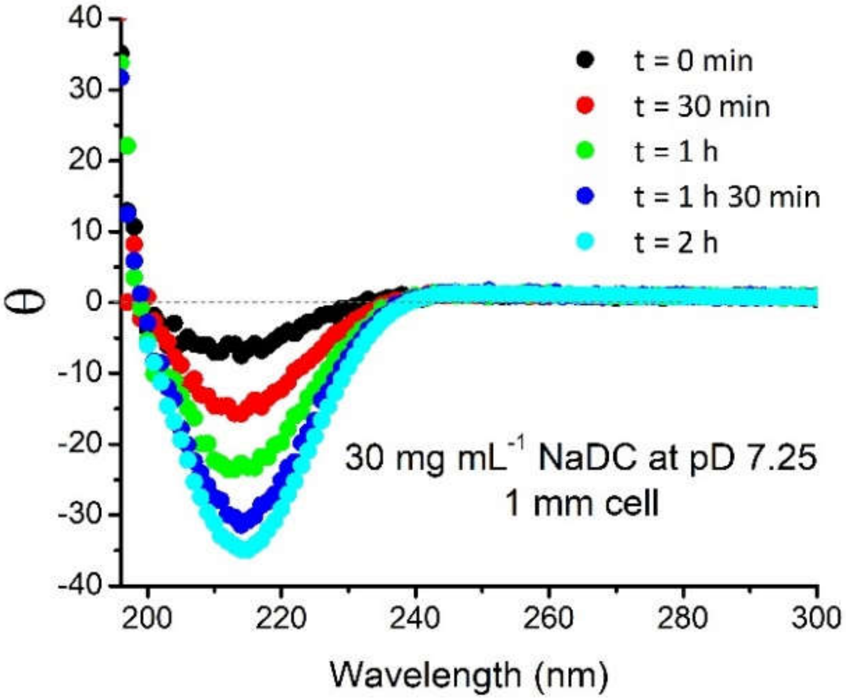

Figure S9. CD spectra of 30 mg mL^{-1} NaDC/water systems in 0.1 M NaCl and pH 7.30 as a function of time.

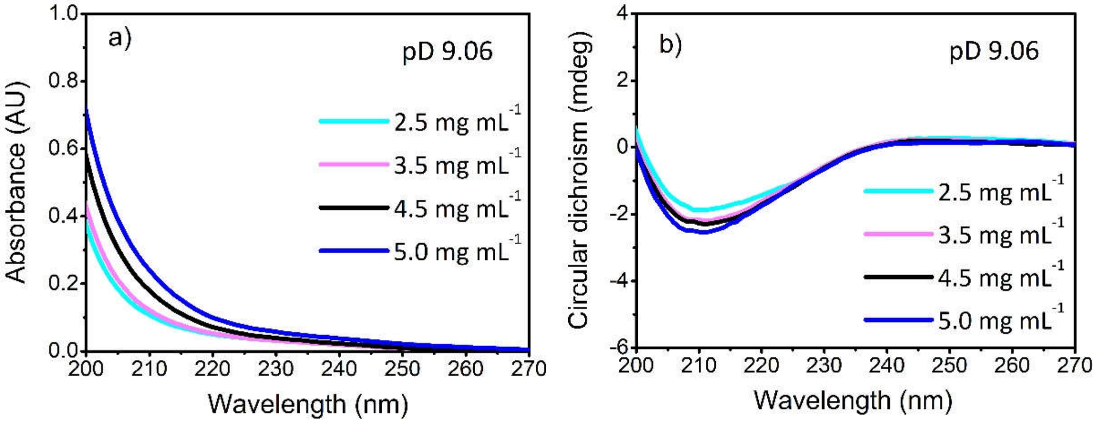

Figure S10. Concentration-dependence of: panel (a) UV-vis and panel (b) CD spectra of NaDC/water systems, recorded at pD 9.06 in quartz-cell of 1 mm path length.

Small Angle Neutron Scattering Experiments

Small-angle neutron scattering (SANS) experiments were performed on the Larmor diffractometer at the ISIS Spallation Neutron Source, Rutherford Appleton Laboratory, Didcot, UK.

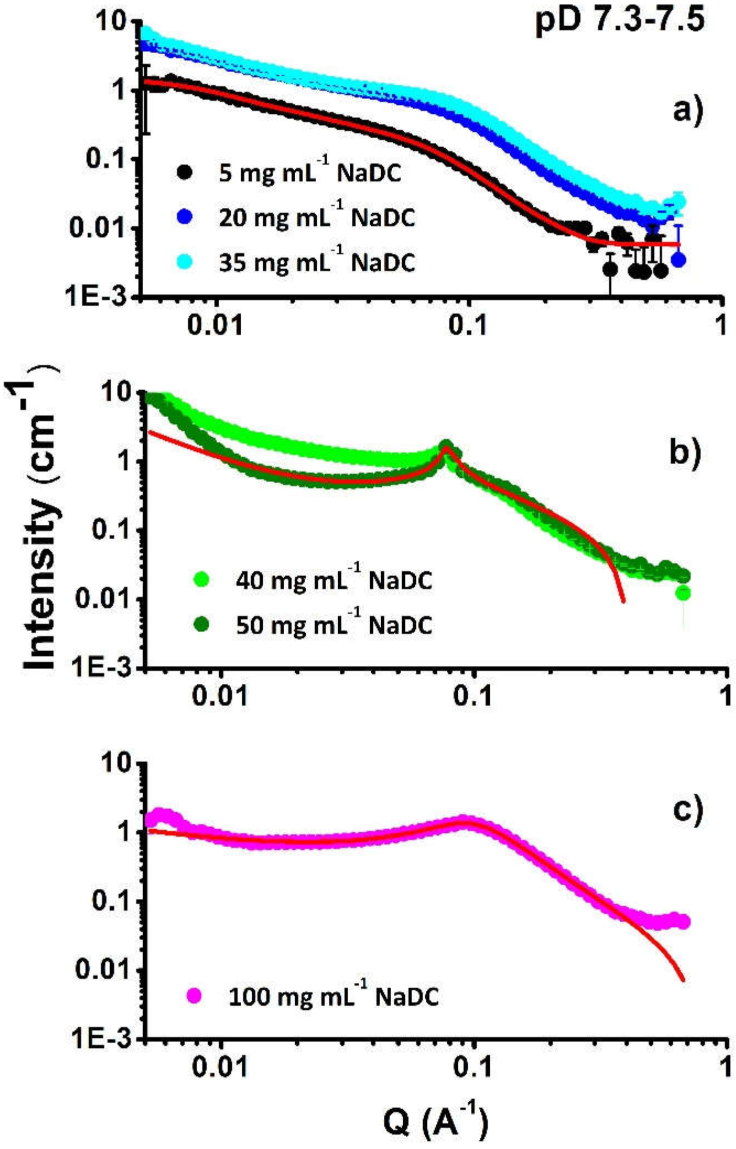

Figure S11. NaDC scattering data as a function of concentration, at pD 7.3 - 7.5. Mathematical fittings were reported as red lines.

Table S6. Parameters obtained for NaDC/water using the hydrated ellipsoid mathematical model: scattering length density (SLD), polar and equatorial radii and charge. Standard deviation is lower than 1%, except where indicated otherwise.

C_{NaDC} (mg mL^{-1})	SLD (10^{-6} Å^{-2})	R_{polar} (Å)	$R_{equatorial}$ (Å)	Charge (e)
5	1.5 ± 0.1	23 ± 1	9	21 ± 3
20	1.4	24	10	15
35	1.7	24	10	16
40	2.2	25	10	15
50	2.7	26	11	14
100	3.1	28	10	13

Table S7. Parameters obtained for 35 mg mL^{-1} NaDC at pD 8.50 - 9.50 in presence of 3.5, 35 or 70 mg mL^{-1} *D*-ARGO7: scattering length density (SLD), polar and equatorial radii and charge. All values have a standard deviation lower than 1 %, except where stated otherwise.

C_{NaDC} (mg mL^{-1})	$C_{D\text{-}ARGO7}$ (mg mL^{-1})	Mathematical Model	SLD (10^{-6} Å^{-2})	R_{polar} (Å)	$R_{equatorial\ major}$ (Å)[a]	Charge (e)
35	-	Hydrated Ellipsoid	1.85	22	9	14
35	3.5	Hydrated Ellipsoid	2.12	28	8	12
35	35	Debye Gaussian coil	-	-	-	-
35	70	Debye Gaussian coil	-	-	-	-

[a] Defined as equatorial major radius in hydrated triaxial ellipsoids, whereas it is defined as equatorial radius in ellipsoid.
